# Supplementary material for: PARP Inhibitors in Brain Metastases from Epithelial Ovarian Cancer through a Multimodal Patient Journey: Case Reports and Literature Review
Source: Int J Mol Sci. 2024 Jul 18;25(14):7887. doi: 10.3390/ijms25147887 (PMC11276770; doi:10.3390/ijms25147887)
Supplement: Supplementary file 1 [file ijms-25-07887-s001.zip › ijms-3095641-supplementary.pdf]

Supplementary data

Table S1. Summary of the available evidence about PARPi use in patients with BMs from EOC, according to the search criteria reported in the *search strategy section (below).*

Author (Year)	Number of cases/ Primary cancer	Age	BRCA mutation status	CNS site(s)	PARPi agent	PARPi setting at time of BMs	Extra-cranial site(s)	Local therapies for BMs	CNS BOR and PFS on PARPi
**Tao [18] (2020)**	1 (EOC)	62	BRCA2 PV	Multiple (fronto-parietal, right cerebellar) BMs	Niraparib	PSR	Abdominal lymph nodes	RT (WBRT)	CR PFS 15 m
**Gray [26] (2019)**	1 EOC	68	BRCA1 PV	Multiple (the largest in the left centrum semiovale) BMs	Niraparib	PSR	No	RT (WBRT)	PR PFS 17 m
**Cabitza [10] (2023)**	1 EOC	47	BRCA1-2 wild-type	Single (left cerebellar) BM	Niraparib	Frontline	No	Surgery	CR PFS 20 m
**Alizzi [5] (2023)**	39 EOC	65 (44–84)	BRCA1-2 PV and wild-type	≥3 (n=25) 1-2 (n=14) BMs	Niraparib Olaparib	PSR	No (n=14) Yes (n=25)	Surgery RT (SRS, WBRT)	CR-PR PFS 18-20 m
**Zhang [32] (2023)**	1 EOC	48	BRCA1-2 wild-type	Single (left frontal) BM	Niraparib	PSR	No	Surgery	CR PFS 29 m
**Kasher-man [15] (2020)**	1 EOC	47	BRCA1 PV	Multiple (right frontal; left cerebellar) BMs	Olaparib	PSR (beyond CNS oligo-recurrence)	No	Surgery and LITT	PR PFS 21 m
**Cerda [41] (2022)**	1 (out of 58 pts) EOC	NR	BRCA1-2 wild-type	≤3 CNS sites	Olaparib	PSR	Peritoneal (≤3 sites)	Surgery RT (SRS)	PR PFS NR

**Gallego [14] (2020)**	1 EOC	54	BRCA1 PV	Multiple (left occipital; right cerebellar) BMs	Olaparib	PSR	Mediasti-nal sites	RT (WBRT)	PR PFS 42 m
**Bangham [34] (2020)**	1 EOC	61	BRCA2 PV	Multiple (left parietal, lepto-meningeal) BMs	Olaparib	PSR (beyond CNS oligo-PD)	No	Surgery RT (SRS)	PR PFS 12 m
**Sakamo-to [35] (2018)**	1 PPC	58	BRCA1 PV	Multiple BMs	Olaparib	PSR	No	None	PR PFS 18 m
**Favier [38] (2020)**	1 EOC	54	BRCA2 PV	Lepto-meningeal BMs	Olaparib	PSR	Peritoneal sites	RT (WBRT)	PR PFS 14 m
**Wang [33] (2021)**	1 G3 USC	54	BRCA1 PV	Multiple (parieto-occipital and right cerebellar) BMs	Niraparib	PSR	No	None	PR PFS 8 m
**Sliwinska [37] (2023)**	5 EOC	48.9 (mean) ±10.8 (SD)	BRCA1-2 PV	Single and multiple BMs	Olaparib	PSR (even beyond CNS-PD)	No	Surgery RT (SRS) (n=2) Palliative CNS RT (n=2)	PR PFS 8 m

Abbreviations. EOC: epithelial ovarian cancer. PPC: primary peritoneal cancer. USC: uterine serous carcinoma. PV: pathogenic variant. BMs: brain metastases. CNS: central nervous system. PSR: platinum-sensitive relapse. BOR: best overall response. CR: complete response. PR: partial response. PFS: progression-free survival. pts: patients. LITT: Laser interstitial thermal therapy. NR: not reported.

Search Strategy

The Literature searches for the present narrative review were conducted in the following databases from 2013 to March 2023: PubMed/MEDLINE; Google Scholar; US National Institutes of Health Ongoing Trials Register. We used the following search terms: (("PARP inhibitor" OR "PARP inhibitors" OR "Niraparib" OR "Olaparib" OR "Rucaparib") AND ("brain metastases" OR "CNS metastases" OR "intracranial recurrence") AND ("ovarian cancer" OR "epithelial ovarian cancer" OR "ovarian carcinoma")). A PubMed (PubMed, RRID:SCR_004846) search alert was used to capture additional articles published between May 2023 and December 2023. Searches were restricted to "epithelial ovarian cancer," "brain metastases", "CNS recurrence" and "PARP inhibitors". A literature search from ASCO Annual Meeting 2024 was performed to capture the ongoing PARPi clinical trials. The 43 articles retrieved from the above sources included preclinical studies, in vitro studies, case reports, case series, retrospective studies and review articles providing a rationale for chemoresistance and potential targeting therapeutics. Only English language articles were included in the searches. Forward citation searching of the reference lists of the research studies and review papers was also performed. Thus, key papers were included based on the authors' clinical experience and knowledge of the field. The results of our research are summarized in Table S1.
